# Supplementary material for: Netcombin: An algorithm for constructing optimal phylogenetic network from rooted triplets
Source: PLoS One. 2020 Sep 18;15(9):e0227842. doi: 10.1371/journal.pone.0227842 (PMC7500971; doi:10.1371/journal.pone.0227842)
Supplement: S4 File — (ZIP) [file pone.0227842.s004.zip › SequenceData-Triplet/sequenceData-10-20-30-0-Triplet/length 400, numseq 40, rate 10^-7/3 seq.pdf]

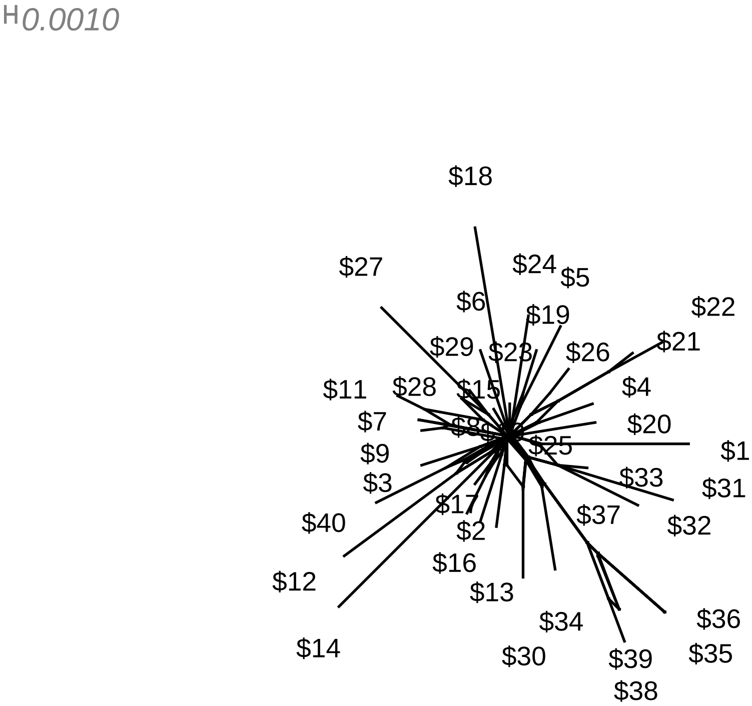
0.0010
$18
$27
$24
$5
$6
$19
$22
$21
$29
$23
$26
$4
$11
$28
$15
$7
$20
$25
$1
$9
$33
$31
$3
$17
$37
$32
$40
$2
$16
$12
$13
$34
$36
$30
$39
$35
$14
$38
